# German Statistics Regarding Anæsthetics

**Published:** 1897-05-08

**Authors:** 


### German Statistics Regarding Anæsthetics.

At the congress of German surgeons held in Berlin in April, Herr Gurlt gave the totals derived from the reports which had been received for the past two years. These reports numbered 85, with 58,769 narcoses. Chloroform had been given about 27,000 times, with 29 deaths; ether, 19,000 times with 3 deaths; morphia, chloroform, and ether, 5,000 times with no death; bromic ether, 996 times with no death; and chloroform and ether, 5,890 times with no death. The proportion of deaths to narcoses during the last two years was one death in 1,836 narcoses. From the commencement of the collection of the statistics in 1891 the narcoses reported amounted to 327,593 with 134 deaths, or one in 2,444 cases, and the anæsthetics, as judged by these cases, might be placed in the following order in regard to danger: pental, 1 death in 213 narcoses; chloroform, 1 in 2,039; Billroth's mixture, 1 in 3,897; ether, 8 in 5,019; bromic ether, 1 in 5,228; chloroform and ether, 1 in 7,591. It is interesting to note that there seems to have been a considerably higher proportionate mortality during recent years, especially in regard to the administration of chloroform. It was decided to continue the inquiry for another year.

